# Psychosocial factors in adults with chronic kidney disease: characteristics of pilot participants in the Tasmanian Chronic Kidney Disease study

**DOI:** 10.1186/1471-2369-14-83

**Published:** 2013-04-12

**Authors:** Charlotte M McKercher, Alison J Venn, Leigh Blizzard, Mark R Nelson, Andrew J Palmer, Michael A Ashby, Jennifer L Scott, Matthew D Jose

**Affiliations:** 1 Menzies Research Institute Tasmania, University of Tasmania, Hobart, Tasmania, Australia; 2 Royal Hobart Hospital, Hobart, Tasmania, Australia; 3 School of Psychology, Faculty of Health Sciences, University of Tasmania, Hobart, Tasmania, Australia; 4 School of Medicine, University of Tasmania, Hobart, Tasmania, Australia

**Keywords:** Anxiety, Chronic kidney disease, Cohort, Depression, Health-related quality of life, Social support

## Abstract

**Background:**

Psychosocial factors including depression, anxiety and lower social support are common in patients with chronic kidney disease (CKD). However the influence of these potentially modifiable risk factors on morbidity and mortality in this renal population is unknown. The Tasmanian Chronic Kidney Disease study is a prospective cohort study which aims to examine the influence of both biomedical and psychosocial factors on disease progression, decision making and length and quality of life in adults with severe CKD, prior to kidney replacement therapy (KRT). This paper describes the recruitment, baseline characteristics and initial follow-up of pilot participants.

**Methods:**

Adults aged > 18 years with stage 4 CKD (eGFR 15–29 mls/min/1.73 m^2^) and not receiving dialysis were recruited via treating physicians. Measures included depression (9-item Patient Health Questionnaire), anxiety (Beck Anxiety Inventory) and social support (Multidimensional Scale of Perceived Social Support). Primary outcomes were kidney disease progression, use of KRT and health-related quality of life (Kidney Disease and Quality of Life Short Form and the EQ-5D).

**Results:**

Of those invited (n = 105), 49 provided consent and completed baseline assessment. There were no significant differences between responders and non-responders in age, gender and socio-economic status (all p > 0.05). Participants were predominantly male (63.3%) with a mean age of 72.6 ± 10.2 years. Mean serum creatinine was 241 ± 62 μmol/L with mean eGFR 22 ± 5 mls/min/1.73 m^2^. Prevalence of major depression and moderate to severe anxiety was 10% and 9% respectively. Less severe depression and fewer anxiety symptoms were associated with higher health-related quality of life. Follow-up at 10-months showed CKD progression in 34% of participants (use of KRT in 16%, stage 5 CKD without KRT in 18%), one death, with the remainder stable at CKD stage 3 or 4.

**Conclusions:**

Results indicate that a larger prospective study is feasible and has the capacity to examine the influence of biomedical and psychosocial factors on kidney disease progression, use of dialysis and transplantation, and salient personal and economic outcomes. Findings have the potential to provide an evidence base for revising healthcare provision in order to optimize the care of patients with CKD.

## Background

Chronic kidney disease (CKD) is a progressive disease associated with decreased quality of life, progression to end-stage kidney disease (ESKD), and increased risk of cardiovascular disease and mortality. Evidence from various chronic disease settings indicates that psychosocial factors including depression, anxiety and social isolation have a significant impact on quality of life, biological factors and disease progression [1-3]. Targeted psychosocial interventions have been shown to improve quality of life, treatment compliance, physical functioning and reduce the risk of cardiovascular disease [1,4]. However, despite the increasing social and economic burden of CKD [5], our understanding of the influence of psychosocial factors on patients in this population is limited. Examining the influence of potentially modifiable psychosocial factors on the progression of disease and management of those with advanced CKD has the capacity to identify areas for psychosocial intervention in this vulnerable and growing population.

Research examining psychosocial factors in renal populations is relatively new and has focussed predominantly on patients with ESKD. Depression is associated with increased mortality and decreased quality of life in patients on dialysis [6-8], however few studies have examined the influence of depression on disease progression and adverse outcomes in patients with CKD prior to the initiation of kidney replacement therapy (KRT) (dialysis or transplantation) [9,10]. These studies indicate that clinical depression [9] and elevated depressive symptoms [10] are independent predictors of clinical outcomes, including initiation of dialysis, hospitalization or death in CKD patients. In contrast, Kop and colleagues found that higher depressive symptoms were associated with subsequent acute kidney injury but not renal function decline, hospitalization or initiation of dialysis [11].

In comparison to depression, there is a paucity of research examining the influence of anxiety and social isolation in patients with kidney disease. Anxiety disorders are often perceived to represent symptoms of depression rather than independent conditions and therefore have been relatively understudied in this medical population. There is a high prevalence of anxiety in patients treated with hemodialysis [12] however the impact of anxiety in CKD patients not on dialysis is unknown. Finally, while lower perceived social support is associated with decreased quality of life [13,14] and increased mortality in ESKD [13-15] this relationship has not been investigated in CKD prior to KRT.

The aim of the current pilot study is to examine the feasibility and efficacy of the Tasmanian Chronic Kidney Disease study, a prospective cohort study of non-dialyzed adults with severe CKD living in Tasmania, Australia [16] with a minimum follow-up of 3-years. The main objectives of this study are to examine the relative influence of both biomedical and psychosocial factors on kidney disease progression, use of KRT and overall length and quality of life. Findings will have direct translation into clinical practice (by identifying psychosocial factors as important predictors of outcomes), healthcare service provision (by focusing health services on quality of life rather than extending life at all costs) and healthcare policy. The current paper describes the study design, the key clinical and psychosocial characteristics of pilot participants at baseline, and clinical progression at 10-month follow-up.

## Methods

### Inclusion criteria and recruitment

All adults living in Tasmania aged > 18 years with an estimated glomerular filtration rate (eGFR) 15–29 mls/min/1.73 m^2^ on a single reading in the previous 3-months and not receiving dialysis between 1 July 2010 and 9 August 2011 were eligible for inclusion in the pilot study. Potential participants were identified by use of the Clinical Audit Tool [17] or through a direct request to pathology services to identify any patient with an eGFR 15–29 mls/min/1.73 m^2^ for the first time during the recruitment period. Recruitment from primary care was irrespective of (and separate to) referral to a renal physician. Eligible participants were sent an enrolment package in the mail by their treating doctor. This consisted of an information booklet and a letter requesting return of a signed consent form indicating participation. If consent forms were not returned within two weeks potential participants were followed-up by phone. The pilot study was approved by the Tasmanian Health and Medical Human Research Ethics Committee (protocol H0011543).

### Procedure

Participants were sent self-report questionnaires prior to attending a study clinic where baseline clinical and laboratory data including height, weight, blood pressure and hemoglobin were measured and functional capacity was assessed. Participants unable to attend a clinic had measurements taken at their place of residence. Clinic assessments took approximately 1.5 hours.

### Measures

#### Psychosocial and sociodemographic factors

Psychosocial factors were self-reported using the Patient Health Questionnaire [18], the Beck Anxiety Inventory [19] and the Multidimensional Scale of Perceived Social Support [20]. All measures have previously been validated in people with kidney disease and were used to facilitate comparisons within the literature. The Beck Anxiety Inventory was included in order to distinguish symptoms of anxiety from those of depression.

##### Patient Health Questionnaire (PHQ-9)

The PHQ-9 is a 9-item screening and diagnostic tool devised to assess the presence and severity of depression. Respondents rate how often they have experienced depressive thoughts or feelings over the prior 2-weeks on a scale ranging from 0 (not at all) to 3 (nearly every day) with total scores ranging from 0 to 27. Scores ≥ 10 have sensitivity and specificity of 92% for a diagnosis of major depression in patients on hemodialysis [21].

##### Beck Anxiety Inventory (BAI)

The BAI is a 21-item screening tool designed specifically to distinguish symptoms of anxiety from those of depression. It also includes an item examining fear of death (thanatophobia) which may influence decision making concerning treatment. Respondents rate the extent to which each symptom has bothered them in the past month, ranging from 0 (not at all) to 3 (severely, I could barely stand it). Total scores range from 0 to 63 with scores of ≥ 16 indicating moderate to severe levels of anxiety [22]. The BAI has demonstrated high internal consistency and adequate discriminant validity in older patients in primary care [23].

##### Multidimensional Scale of Perceived Social Support (MSPSS)

The MSPSS is a 12-item inventory which assesses overall perceived social support. The scale contains three subscales, family, friends, and significant others, which are averaged to comprise an overall score. Scores are reported on seven-point scale, with higher scores indicating higher perceived social support. Lower levels of perceived social support derived using this scale have predicted poorer survival in hemodialysis patients [24].

Sociodemographic and health information including marital status, education level, employment status, household income and smoking history was collected via questionnaire. Area-level socio-economic status (SES) based on residential postcode was derived using the Australian Bureau of Statistics socioeconomic index for areas (SEIFA) [25]. The SEIFA is a summary of four indices designed to measure different aspects of SES based on questions asked in the 2001 Census of Population and Housing [25]. The Index of Disadvantage is derived from attributes including low income, low educational attainment, high unemployment and employment in unskilled occupations. For the current analysis postcodes were divided into four quartiles according to ranking in this index, the first quartile representing greater disadvantage compared with other geographical areas.

#### Biomedical factors

Information regarding the number and severity of medical comorbidities was obtained from the participant’s treating doctor using a modified Charlson comorbidity index (CMI) [26]. Body mass index (BMI) (kg/m^2^) was derived from objectively measured height and weight and categorized according to standard BMI cut-points [27]. Cognitive function was assessed objectively using the Modified Mini-Mental State Examination [28] and via self-report using the cognitive function subscale of the Kidney Disease Quality of Life short-form (KDQOL-SF 1.3) [29]. Functional status was reported by the participant’s treating doctor using the Karnofsky Performance Scale [30] while functional ability was self-reported using the Activities of Daily Living index [31] and the Instrumental Activities of Daily Living scale [32]. Functional capacity was assessed using hand grip dynometry (average of two measurements) and gait speed (time taken to walk 5-metres) using standardized protocols. Healthcare decision making was self-reported using the Autonomy Preference Index [33] and the Krantz Health Opinion Survey [34].

##### Modified Mini-Mental State Examination (3MS)

The 3MS is an interviewer administered test of global cognitive function which assesses orientation, attention, language, and memory. Scores range from 0 to 100 with higher scores indicating better cognitive function. The 3MS is more sensitive to early cognitive changes than the mini-mental state with scores < 80 having a sensitivity of 97% and a specificity of 91% for detecting dementia [28].

##### Kidney Disease Quality of Life short-form (KDQOL-SF 1.3)

The KDQOL-SF combines the 36 generic items of the SF-36 with 43 kidney disease-targeted items. Eleven subscales are defined from the kidney disease-targeted items, resulting in a total of 19 subscales (i.e., eight generic and 11 targeted to patients with kidney disease and treated by dialysis). Responses are weighted and transformed to scores ranging from 0 to 100, with higher scores indicating better self-assessed health-related quality of life. The SF-36 has two subscales, the physical component summary (SF-36 PCS) and the mental component summary (SF-36 MCS). The KDQOL-SF 1.3 cognitive function subscale has been validated in people with CKD with a median score of 87 [35].

##### Karnofsky Performance Scale (KPS)

The KPS is a physician-rated measure of a patient’s functional status. Scores range from 0 to 100 with a score of 100 indicating full capacity to carry out normal activities. Scores < 70 indicate that some level of assistance is needed to carry out daily activities. Lower KPS scores have predicted higher risk of mortality in patients with ESKD [36].

##### Activities of Daily Living (ADL)

The ADL assesses functional status as a measure of the patient’s ability to perform activities of daily living independently. Scores range from 0 to 6 with a score of 6 indicating full function. The scale is sensitive to changes in declining health status and has been used effectively among older adults in a variety of care settings.

##### Instrumental Activities of Daily Living (IADL)

The IADL assesses independent living skills in order to identify current functioning as well as improvement or deterioration over time. Patients are scored according to their highest level of functioning across 8 domains with a summary score ranging from 0 (low function, dependent) to 8 (high function, independent).

##### Autonomy Preference Index (API)

The API identifies dimensions of autonomy by measuring patients’ preferences for making medical decisions (decision-making subscale) and their desire to be informed (information-seeking subscale). Higher scores indicate stronger preferences in favor of decision making or information-seeking.

##### Krantz Health Opinion Survey (KHOS)

The KHOS measures preferences regarding information about healthcare (information-seeking subscale) and active involvement in healthcare (behavioral-involvement subscale). While the API measures preference for healthcare information-seeking behavior for decisions not necessarily already made, the KHOS examines manifest information-seeking and behavioral involvement. Higher scores indicate that the patient is proactive in seeking healthcare information and professional medical assistance.

#### Outcome measures

Primary outcome measures were kidney disease progression, use of KRT and subjective health-related quality of life. Kidney disease progression was measured continuously according to decrease in mean eGFR and categorically per the Kidney Disease Outcomes Quality Initiative staging system [37] using the Modification of Diet in Renal Disease formula [38]. Use of KRT was obtained by consented linkage to the Australia and New Zealand Dialysis and Transplant Registry (ANZDATA). Health-related quality of life was assessed using both a psychometric health status measure (KDQOL-SF 1.3) and a utility-based measure (EQ-5D) [39]. Health status measures discriminate levels of functioning between groups and detect changes in function over time while utility-based measures are utilized in cost-effectiveness analyses and to guide decisions regarding resource allocation. The EuroQol Group’s EQ-5D is commonly used to calculate quality adjusted life years (QALYs) [40] and has been used extensively to assess utility-based quality of life in CKD patients [41]. The EQ-5D has five dimensions (mobility, self-care, usual activities, pain/discomfort, and anxiety/depression). In the current analysis responses to the EQ-5D 3-level version were dichotomized as ‘no problem’ versus ‘some/severe problems’.

### Statistical analysis

Data are presented descriptively as mean (standard deviation) or median (range) for continuous variables, and frequency (percentage) for categorical variables. Between-group differences were compared with Student *t* test or the chi-square test, where appropriate. Associations between psychosocial and clinical variables with health-related quality of life were calculated using Spearman’s correlation coefficient. Analyses were conducted using Stata/IC version 12.0 (Statacorp, 2011) with statistical comparison treated as significant at α = 0.05 (two-tailed).

## Results

**Figure 1:**
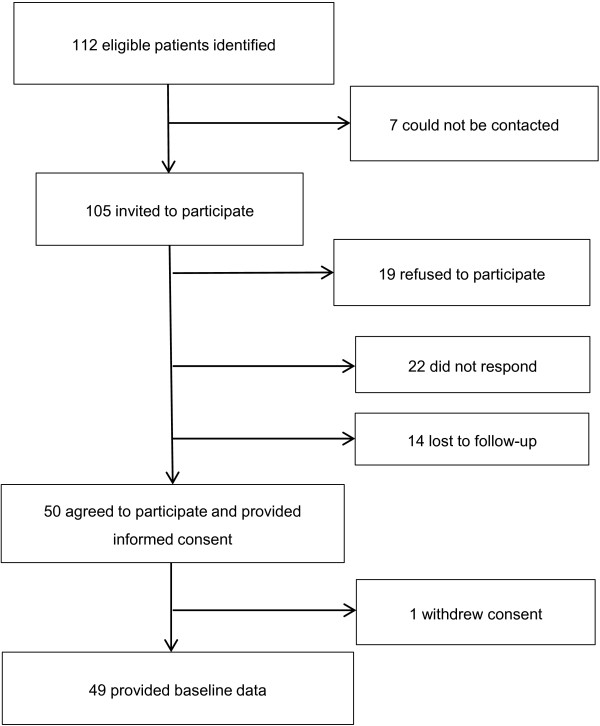
Description of the Tasmanian Chronic Kidney Disease study pilot cohort.

An overview of recruitment and retention is presented in Figure 1. Of the 105 eligible patients approached to participate, 18% (n = 19) declined participation, 21% (n = 22) did not respond and 13% (n = 14) were lost to follow-up. Subsequently 48% (n = 50) of those approached provided informed consent and 47% (n = 49) completed baseline assessments. There were no significant differences between responders and non-responders in mean age, gender distribution and Index of Disadvantage quartiles (all p > 0.05).

The baseline sociodemographic, clinical and laboratory characteristics of pilot participants are displayed in Table 1. In summary, participants were predominantly male with a mean age at enrolment of 72.6 ± 10.2 years (range 37–86 years). The majority had low educational attainment, were married or living as married and were receiving a Government pension. Around 70% of participants reported a household income of less than AUS$31,200 per year. Only three participants were current smokers, while around half (n = 24) were former smokers and over 75% were overweight or obese. Around 60% of participants had at least three current medical comorbidities and 39% had at least four comorbidities. The majority of participants were hypertensive and around 40% had diabetes mellitus.

**Table 1:** Baseline characteristics of pilot participants, the Tasmanian Chronic Kidney Disease study (frequency (%) unless otherwise indicated)

**Variable**	
*Sociodemographic*	
Age (yr; mean ± SD) [range]	73 ± 10 [37–86]
Female gender	18 (37%)
Education > Year 12	12 (25%)
Married/living as married	34 (69%)
Retired	35 (71%)
Government pension	35 (71%)
Index of Disadvantage	
Quartile 1	13 (26%)
Quartile 2	13 (26%)
Quartile 3	10 (20%)
Quartile 4	14 (28%)
Household income (AUS$ annual gross)	
1–10,399	1 (2%)
10,400–15,599	6 (12%)
15,600–20,799	10 (20%)
20,800–31,199	17 (35%)
31,200–41,599	2 (4%)
41,600–51,999	1 (2%)
52,000–64,999	2 (4%)
65,000–103,999	0 (0%)
≥104,000	2 (4%)
Declined to answer	8 (16%)
*Clinical*	
Depression (PHQ-9 score ≥ 10)	5 (10%)
Anxiety (BAI score ≥ 16)	4 (9%)
Current/former smoker	27 (55%)
≥3 medical comorbidities	29 (59%)
No. of comorbidities (mean ± SD) [range]	3.3 ± 1.9 [1.0–8.0]
Hypertension	41 (84%)
Systolic BP (mm Hg; mean ± SD)	133.3 ± 22
Diastolic BP, (mm Hg; mean ± SD)	67.8 ± 12.7
Diabetes mellitus	19 (39%)
Atherosclerotic heart disease	21 (43%)
Congestive heart failure	17 (35%)
Peripheral vascular disease	14 (29%)
Grip strength, right (kg; mean ± SD) [range]	25.9 ± 8.6 [9.0–49.0]
Grip strength, left (kg; mean ± SD) [range]	26.9 ± 8.8 [12.5–47.5]
5-metre walk (sec; mean ± SD) [range]	8.0 ± 4.4 [5.0–28.0]
BMI (kg/m^2^; mean ± SD) [range]	30.1 ± 6.8 [21.9–53.5]
BMI < 25 kg/m^2^	8 (16%)
BMI 25–29.9 kg/m^2^	23 (47%)
BMI ≥ 30 kg/m^2^	15 (31%)
*Missing*	*3 (6%)*
*Laboratory*	
Hemoglobin (g/L; mean ± SD) [range]	121.5 ± 15.0 [94.0–163.0]
Serum creatinine (μmol/L; mean ± SD) [range]	241.1 ± 61.9 [144.0–396.0]
eGFR (mL/min/1.73 m^2^; mean ± SD) [range]	22.2 ± 4.9 [15.0–30.0]

Mean depression (PHQ-9) score was 4.4 ± 5.8 with five participants (10%) meeting the recommended threshold (score ≥ 10) for a clinical diagnosis of major depression. Mean anxiety (BAI) score was 7.8 ± 7.8 with 9.3% of participants reporting at least moderate levels of anxiety (score ≥ 16). Mean social support (MSPSS) score was 5.8 ± 1.1 (range 2.6–7.0).

Baseline scores for the health-related quality of life measures (KDQOL-SF 1.3 and EQ-5D) are shown in Table 2. Mean values for the kidney disease targeted-scales ranged from 33.7 (work status, SD = 31.3) to 87.4 (social support, SD = 21.1) on the range of total possible (0–100) scores. Mean values for the SF-36 scales ranged from 42.7 (role limitations–physical, SD = 41.9) to 79.6 (social function, SD = 25.9). The highest percentage of problems reported by participants using the EQ-SD was in the dimension pain/discomfort (61%) followed by mobility (51%), usual activities (49%) and anxiety/depression (27%).

**Table 2:** Baseline descriptive statistics for health-related quality of life measures (KDQOL-SF 1.3 and EQ-5D), the Tasmanian Chronic Kidney Disease study

**Variable**	
**Kidney disease-targeted scales**	**(mean ± SD) [range]^**
Burden of kidney disease	71.9 ± 27.0 [6.3–100.0]
Quality of social interaction	80.3 ± 19.2 [26.7–100.0]
Cognitive function	84.1 ± 21.3 [0.0–100.0]
Symptoms/problems	76.1 ± 15.7 [31.8–100.0]
Effects of kidney disease	86.3 ± 16.4 [14.3–100.0]
Sexual function	65.9 ± 37.1 [0.0–100.0]
Sleep	63.2 ± 20.0 [5.0–95.0]
Social support	87.4 ± 21.1 [0.0–100.0]
Work status	33.7 ± 31.3 [0.0–100.0]
Overall health rating	64.5 ± 19.6 [20.0–100.0]
**SF-36 scales**	
Physical Functioning	52.1 ± 29.6 [0.0–100.0]
Role limitations-physical	42.7 ± 41.9 [0.0–100.0]
Pain	66.1 ± 31.2 [0.0–100.0]
General health	43.8 ± 21.2 [0.0–85.0]
Emotional well-being	76.6 ± 20.3 [8.0–100.0]
Role limitations-emotional	63.2 ± 42.0 [0.0–100.0]
Social function	79.6 ± 25.9 [0.0–100.0]
Energy/fatigue	47.7 ± 24.5 [5.0–90.0]
SF-36 PCS	37.7 ± 10.1 [17.5–55.1]
SF-36 MCS	50.7 ± 9.4 [19.1–64.1]
**EQ-5D**	**% reporting problems**
Mobility	51.0
Self-care	12.2
Usual activities	49.0
Pain/discomfort	61.2
Anxiety/depression	26.5

^No participants were receiving dialysis at time of enrolment so ‘Staff encouragement’ and ‘Patient satisfaction’ scales were not completed.

Associations between psychosocial and biomedical variables, and health-related quality of life subscales (KDQOL-SF) are displayed in Table 3. Consistent inverse associations were observed between depression severity (PHQ-9) and anxiety (BAI) with all of the SF-36 subscales and the majority of kidney disease-targeted subscales. Significant positive correlations were also observed between perceived social support (MSPSS) and the cognitive function and emotional well-being subscales. There were no significant associations between eGFR, hemoglobin, creatinine, and health-related quality of life.

**Table 3:** Spearman correlations between the KDQOL-SF subscales and psychosocial and biomedical variables, the Tasmanian Chronic Kidney Disease study

**KDQOL-SF**	**PHQ-9**	**BAI**	**MSPSS**	**Age**	**Hemoglobin**	**Creatinine**	**eGFR**	**CMI**	**CF**	**ADL**	**KPS**
**Kidney disease-targeted scales**											
Burden of kidney disease	**−0.33**^**a**^	**−0.33**^**a**^	−0.05	**0.29**^**a**^	0.03	−0.13	0.15	0.02	**0.34**^**a**^	0.08	−0.03
Quality of social interaction	**−0.56**^**c**^	**−0.60**^**c**^	**0.45**^**b**^	0.23	0.05	0.17	−0.12	−0.14	**0.58**^**c**^	**0.37**^**b**^	0.12
Cognitive function	**−0.51**^**c**^	**−0.55**^**c**^	**0.47**^**b**^	0.05	−0.16	0.03	−0.06	−0.20	1.00	**0.36**^**a**^	0.21
Symptoms/problems	**−0.61**^**c**^	**−0.61**^**c**^	0.11	0.15	−0.05	−0.07	0.11	−0.16	**0.45**^**b**^	0.21	0.18
Effects of kidney disease	**−0.50**^**c**^	**−0.41**^**b**^	0.13	−0.004	−0.22	−0.09	−0.02	**−0.31**^**a**^	**0.43**^**c**^	0.23	0.17
Sexual function	−0.12	−0.06	−0.08	0.19	−0.18	0.23	−0.26	−0.26	−0.41	0.51	0.28
Sleep	**−0.67**^**c**^	**−0.61**^**c**^	0.27	0.28	0.00	−0.02	0.09	**−0.36**^**a**^	**0.50**^**b**^	**0.30**^**a**^	0.16
Social support	**−0.34**^**a**^	−0.25	**0.54**^**c**^	0.20	−0.18	−0.11	0.01	**−0.36**^**a**^	**0.49**^**c**^	**0.42**^**b**^	0.25
Work status	−0.12	−0.06	0.17	**−0.32**^**a**^	0.04	0.15	−0.21	−0.14	0.09	0.13	0.23
Overall health rating	**−0.54**^**c**^	**−0.44**^**b**^	0.24	0.03	−0.22	0.01	−0.14	**−0.46**^**b**^	**0.36**^**a**^	**0.29**^**a**^	**0.30**^**a**^
**SF-36 scales**											
Physical functioning	**−0.46**^**b**^	**−0.52**^**c**^	0.09	−0.16	0.15	0.05	−0.02	**−0.38**^**a**^	0.23	**0.33**^**a**^	**0.10**^**b**^
Role limitations-physical	**−0.45**^**b**^	**−0.42**^**b**^	−0.06	−0.14	0.00	0.05	−0.03	−0.25	**0.31**^**a**^	0.26	0.23
Pain	**−0.37**^**b**^	**−0.60**^**c**^	0.12	0.08	0.14	0.05	0.01	**−0.30**^**a**^	**0.29**^**a**^	0.20	0.29
General health	**−0.72**^**c**^	**−0.63**^**c**^	0.13	0.19	0.08	0.04	−0.001	**−0.48**^**c**^	**0.30**^**a**^	0.16	0.27
Emotional well-being	**−0.50**^**c**^	**−0.64**^**c**^	**0.41**^**b**^	0.14	0.11	0.24	−0.21	−0.27	**0.59**^**b**^	**0.32**^**a**^	0.15
Role limitations - emotional	**−0.35**^**a**^	**−0.37**^**a**^	−0.11	0.11	0.24	−0.06	0.20	0.14	0.06	−0.03	0.09
Social function	**−0.67**^**c**^	**−0.62**^**c**^	0.18	0.06	0.17	0.09	−0.10	−0.25	**0.57**^**b**^	0.28	0.25
Energy/fatigue	**−0.65**^**c**^	**−0.68**^**c**^	0.22	−0.06	−0.05	0.08	−0.07	**−0.33**^**a**^	**0.40**^**b**^	0.15	**0.34**^**a**^
SF36 physical composite score	**−0.48**^**b**^	**−0.61**^**c**^	0.15	0.08	−0.04	0.15	−0.12	−0.29	0.26	0.18	0.14
SF36 mental composite score	**−0.61**^**c**^	**−0.58**^**c**^	0.26	0.23	0.05	0.22	−0.11	−0.12	**0.47**^**b**^	0.30	0.21

^a^p < 0.05; ^b^p < 0.01; ^c^p < 0.001.

ADL, Activities of Daily Living; BAI, Beck Anxiety Inventory; CF, cognitive function subscale; CMI, Charlson comorbidity index; eGFR, estimated Glomerular Filtration Rate; KPS, Karnofsky Performance Scale index; MSPSS, Multidimensional Scale of Perceived Social Support; PHQ-9, Patient Health Questionnaire.

In regards to cognitive function, mean 3MS score was 90.0 ± 9.2 with scores ranging from 65 to 100. Seven participants (15%) scored below the recommended cut-point for lower global cognitive functioning (score < 80). One participant had a score at the test ceiling and no participants had scores at the test floor. Mean score on the KDQOL-SF cognitive function subscale was 84.1 ± 21.3 with a median score of 93.3 (range 0 to 100). Eighteen (37%) participants had scores at the test ceiling with only one participant scoring at the test floor.

Decision making preferences indicated that the majority of participants (63%, n = 30) prefer to delegate decisions to healthcare professionals (API decision-making subscale, mean score 16.6 ± 4.3) and just under half of participants (46%, n = 22) prefer information/education to be provided (API information-seeking subscale, 33.5 ± 3.3). Conversely, around half (49%, n = 23) reported actively seeking information (KHOS information subscale, 10.7 ± 2.1) with 40% of participants (n = 18) preferentially seeking professional help (KHOS behavioral-involvement subscale, 10.6 ± 1.6).

Mean ADL score was 5.6 ± 1.0 and mean IADL score was 7.2 ± 1.3, both indicating reasonably high functioning. Mean KPS score was 77.6 ± 14.2 with a range of 40 to 90 also indicating reasonable functional status. Eight participants (17.4%) were assigned a score < 70 indicating that these participants require some level of assistance to carry out daily activities.

At 10-month follow-up in April 2012, one participant had died while eight participants (16%) had progressed to KRT and nine participants (18%) had progressed to stage 5 CKD without KRT. The remainder of participants remained stable with stage 3 or 4 CKD. For participants not receiving KRT at 10-month follow-up mean serum creatinine was 288 ± 143 μmol/L with mean eGFR 20 ± 9 mls/min/1.73 m^2^.

## Discussion

The Tasmanian Chronic Kidney Disease Study is a prospective cohort study of community-dwelling non-dialyzed adults with severe CKD. The study will contribute to the emerging international literature investigating associations between psychosocial factors and kidney disease and, to our knowledge, will be the first study to examine the influence of these factors in adults with CKD prior to dialysis or transplantation. The current analysis describes the clinical, sociodemographic, psychosocial and health-related quality of life characteristics of pilot participants at baseline and their clinical progression at 10-month follow-up. Strengths include the use of a community-based cohort and comprehensive assessment of a range of psychosocial and biomedical variables.

Considerable psychological morbidity was observed in pilot participants with 10% meeting the threshold for a diagnosis of major depression and 9% reporting at least moderate levels of clinical anxiety. While numbers are small, this is substantially higher than the 12-month prevalence of affective and anxiety disorders (4.6% and 5.2% respectively) observed in Australians aged 65 to 85 years [42]. Consistent with previous research, less severe depression was associated with higher subjective health-related quality of life [43]. Lower levels of anxiety were also associated with higher quality of life suggesting that anxiety symptoms may be associated with quality of life independent to those of depression. Anxiety and depression are distinct clinical entities that have differing causes, pathophysiological processes, and treatments [44]. Distinguishing between their individual and combined effects will be important to understanding the mechanisms by which psychological states are associated with kidney function. While perceived social support (MSPSS) was comparable to population norms [45], participants rated the *quality* of their social interactions (KDQOL-SF) similarly to patients on dialysis [29].

Substantial loss of physical well-being was also observed in pilot participants with self-reported physical functioning, general health and energy/fatigue (KDQOL-SF) being comparable to patients on dialysis [29]. Similarly, all SF-36 subscale scores, except for the emotional well-being and the mental component summary scores, were lower than the age and sex standardized mean scores in the Australian population [46]. Further, over 60% of participants reported experiencing pain/discomfort and around 50% endorsed problems with mobility and usual activities, further highlighting the considerable physical symptom burden experienced by current participants. As previously observed, there were no significant associations between renal function parameters and health-related quality of life [47]. A possible explanation is that while the disease burden in current participants is substantial they may deny the impact of their illness as a coping mechanism.

There are several limitations of the current investigation that should be considered. As this study was designed as a pilot to assess the feasibility of a larger cohort study a formal power calculation was not performed. Results should therefore be treated as preliminary and interpreted with caution. In addition, due to the cross-sectional design, the direction of causality cannot be inferred. Further, depressive and anxiety symptoms were ascertained via self-report rather than standardized clinical interviews which may underestimate the prevalence of these disorders [18,48]. However all self-report measures used in the current study have been validated in renal populations and are brief and easy to administer and interpret. Finally, although non-participants did not differ from participants in terms of age, gender and socio-economic status, the potential for non-response bias remains.

While the participation rate in the current study appears low, it is comparable to previous pilot studies involving adults with chronic disease [49] and consistent with evidence that those with poorer health status, lower levels of functioning and higher levels of psychological distress are less likely to participate in population-based research [50]. Our aim is to recruit 700 participants for the state-wide prospective cohort study. This represents 30% of the target population [16] which is well below the current response rate of 47%. Despite this, efforts to increase participation and limit attrition will be important in ensuring adequate statistical power to examine associations between the key variables of interest. Future strategies involve recruiting face-to-face via treating physicians and assessing participants during usual care thereby increasing response rates and reducing participant burden.

Australia will spend over AUS$11 billion on dialysis and kidney transplantation in the next 10 years [51]. According to Australian registry data, for every new case that receives dialysis another patient with ESKD dies without having received it [52]. Further, for those who do commence dialysis, around 37% will die via withdrawal from treatment [53]. For these patients, medical management may involve a shift from efforts to prolong life to a more supportive/palliative care model focusing on care, quality of life and symptom control. As a patient proceeds from the community to hospital treatment, possible dialysis, then death, there are critical opportunities to improve health and quality of life by identifying and then modifying psychological and social health determinants.

## Conclusions

This paper describes the study design, baseline characteristics and initial follow-up of pilot participants in the Tasmanian Chronic Kidney Disease study. Current results indicate that ongoing recruitment and prospective assessment of this cohort is feasible and has the potential to evaluate the relative impact of both biomedical and psychosocial factors on rate of disease progression, use of KRT, and salient personal and economic outcomes. Findings from this study have the potential to provide an evidence base for revising healthcare decision making and treatment pathways in order to optimize the care of patients with CKD.

## Competing interests

The authors declare that they have no competing interests.

## Authors’ contributions

CM drafted the manuscript, performed the statistical analysis and incorporated critical revisions of the manuscript. AV, CB, MN, AP, MA and JS all contributed to the study design, interpretation of the data and critical revisions of the manuscript. MJ contributed to the study design, acquisition and interpretation of the data, and drafting of the manuscript. All authors read and approved the final manuscript.

## Pre-publication history

The pre-publication history for this paper can be accessed here:

http://www.biomedcentral.com/1471-2369/14/83/prepub
